# Neurodegeneration in multiple sclerosis

**DOI:** 10.1002/wsbm.1583

**Published:** 2022-08-10

**Authors:** Gabrielle M. Mey, Kedar R. Mahajan, Tara M. DeSilva

**Affiliations:** ^1^ Department of Neurosciences Lerner Research Institute, Cleveland Clinic Foundation, and Case Western Reserve University Cleveland Ohio USA; ^2^ Mellen Center for MS Treatment and Research Neurological Institute, Cleveland Clinic Foundation Cleveland Ohio USA

**Keywords:** axonal injury, demyelinating diseases, multiple sclerosis, neurodegeneration, neuroinflammation

## Abstract

Axonal loss in multiple sclerosis (MS) is a key component of disease progression and permanent neurologic disability. MS is a heterogeneous demyelinating and neurodegenerative disease of the central nervous system (CNS) with varying presentation, disease courses, and prognosis. Immunomodulatory therapies reduce the frequency and severity of inflammatory demyelinating events that are a hallmark of MS, but there is minimal therapy to treat progressive disease and there is no cure. Data from patients with MS, *post‐mortem* histological analysis, and animal models of demyelinating disease have elucidated patterns of MS pathogenesis and underlying mechanisms of neurodegeneration. MRI and molecular biomarkers have been proposed to identify predictors of neurodegeneration and risk factors for disease progression. Early signs of axonal dysfunction have come to light including impaired mitochondrial trafficking, structural axonal changes, and synaptic alterations. With sustained inflammation as well as impaired remyelination, axons succumb to degeneration contributing to CNS atrophy and worsening of disease. These studies highlight the role of chronic demyelination in the CNS in perpetuating axonal loss, and the difficulty in promoting remyelination and repair amidst persistent inflammatory insult. Regenerative and neuroprotective strategies are essential to overcome this barrier, with early intervention being critical to rescue axonal integrity and function. The clinical and basic research studies discussed in this review have set the stage for identifying key propagators of neurodegeneration in MS, leading the way for neuroprotective therapeutic development.

This article is categorized under:

Immune System Diseases > Molecular and Cellular Physiology
Neurological Diseases > Molecular and Cellular Physiology

## Graphical abstract

Chronic demyelination results in impaired axon metabolism and function, ultimately leading to progressive neurological decline. Neuroprotective strategies aim to protect axons from inflammatory‐mediated destruction, thereby improving axonal integrity and clinical disability (Image created in BioRender.com).

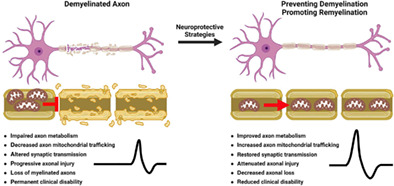

## 1 INTRODUCTION

Multiple sclerosis (MS) is an immune‐mediated chronic inflammatory and neurodegenerative disease of the central nervous system (CNS). MS is a leading cause of disability in young adults and is estimated to affect 2.8 million people worldwide (Walton et al., 2020). The etiology of MS is unclear, but factors associated with disease risk have been identified including geographic location, genetic composition, and biological sex, among others. Regions with the highest prevalence of MS are in North America and Europe, with higher latitudes associated with greater MS prevalence (Simpson Jr. et al., 2019; Walton et al., 2020). More than 230 risk alleles have been identified in genome‐wide association studies, mostly associated with immune system function (International Multiple Sclerosis Genetics Consortium, 2019). In addition, females are two to three times as likely to be diagnosed with MS as males, which suggests a hormonal contribution to disease onset (Walton et al., 2020).

The primary event leading to symptomatic pathology is the infiltration of peripheral immune cells that have been primed against components of the myelin sheath. The predominant hallmark of MS is the development of focal inflammatory and demyelinating lesions that appear in white matter regions of the brain, optic nerve, and spinal cord, although intracortical and deep gray matter lesions are also found. Peripheral immune cells, primarily T and B lymphocytes and macrophages, infiltrate into the CNS parenchyma leading to the formation of (1) perivascular demyelination, (2) subependymal/pial demyelination, and (3) neuroaxonal degeneration (Arrambide et al., 2018; Brownell & Hughes, 1962; Riederer et al., 2019). Inflammatory demyelination and impaired recovery eventually leads to axonal transection that results in permanent neurodegeneration and clinical disability (Losseff, Wang, et al., 1996; Trapp et al., 1998).

There are three main disease courses of MS known as relapsing–remitting (RRMS), primary progressive (PPMS), and secondary progressive (SPMS) MS. Within each clinical course, patients may experience variable disease activity, which is evidenced by new or increasing neurologic dysfunction or the appearance of new/enlarging T2‐weighted gadolinium enhancing lesions on magnetic resonance imaging (MRI), indicative of disease activity (Confavreux et al., 2000; Lublin et al., 2014). An MS diagnosis may be preceded by a symptomatic inflammatory event lasting at least 24 h known as clinically isolated syndrome (CIS). Identification of additional lesions in different regions (juxtacortical/cortical, periventricular, and infratentorial brain and spinal cord) or establishing dissemination in time (by cerebrospinal fluid markers discussed below or presence of MRI gadolinium enhancing and nonenhancing lesions) will establish the diagnosis as MS. (Miller et al., 2005; Thompson et al., 2018). RRMS typically involves periods of clinical relapses coinciding with the appearance of inflammatory lesions with variable recovery. SPMS describes patients who initially had a relapsing course but later have accumulated disability. Both primary and secondary progressive MS disease courses exhibit increasing clinical disability with less frequent inflammatory activity (Confavreux et al., 2000; Lublin et al., 2014). These disease courses are useful in selecting cohorts for clinical trials that may be enriched for disease activity/clinical relapses (RRMS) or gradual disability accumulation (progressive MS trials), and by regulatory agencies (e.g., Food and Drug Administration and European Medicines Agency) in labeling the indications for disease modifying therapies (DMTs). As patients experience inflammation and neurodegeneration throughout their disease course, these labels adequately capture the heterogeneity and complexity of MS pathology. Currently approved DMTs are primarily immunomodulatory by attenuating immune cell proliferation or extravasation into the CNS, which reduces the frequency and severity of inflammatory relapses and improves quality of life. Few pharmacological remedies have proven efficacious for the treatment of progressive disease (Dobson & Giovannoni, 2019). Studies investigating neurodegeneration in MS aim to elucidate markers of neurodegeneration, mechanisms underlying progressive decline, and potential methods to promote remyelination and repair. Inflammatory lesions can cause secondary neurodegeneration characterized by axonal and neuronal loss. Greater T2‐lesion volume is associated with deep gray matter (e.g., thalamic) atrophy and accrual of spinal cord lesions is associated with upper cervical cord atrophy, both predictors of worsened clinical disability (Bischof et al., 2021; Houtchens et al., 2007; Losseff, Wang, et al., 1996; Papadopoulos et al., 2009; Shiee et al., 2012; Zeydan et al., 2018). Data from *post‐mortem* MS tissue and in animal models have proposed several mechanisms of neurodegeneration including a chronic inflammatory milieu, mitochondrial dysfunction, and impaired remyelination capacity. The extent to which these processes occur throughout the disease course and the driving mechanisms behind the variability of recovery following inflammatory relapses in patients is not clear. This review will discuss several neuropathological characteristics of MS, highlighting prevalent features that are indicative of disease progression and the strides that have been made both in MS and in animal models of disease to understand and improve neurodegeneration and neuroprotection.

## 2 IMAGING AND PATHOLOGICAL HALLMARKS OF MULTIPLE SCLEROSIS

The first cases of MS were defined in the 1860s by Jean Martin Charcot and Alfred Vulpian with the observation of sclerotic lesions in the white matter of the spinal cord. This “sclérose en plaques” was further defined in a series of lectures by Charcot, including clinical observations and microscopic descriptions of lesions from spinal cord samples (Zalc, 2018). One of the hallmarks of MS today remains the presence of focal demyelinating lesions, which can be found throughout the brain, optic nerve, and spinal cord (Brownell & Hughes, 1962; Trip & Miller, 2005). The combination of MRI techniques and histopathological examination of biopsies and *post‐mortem* tissue has led to the identification of multiple disease subtypes, lesion classifications, and neurodegenerative features that have been used to define and predict MS clinical course and prognosis.

### 2.1 Clinical and molecular biomarkers

#### 2.1.1 Diagnosing MS

In a patient who has experienced a typical demyelinating event, MRI is effective in identifying the dissemination of lesions in space and time (Thompson et al., 2018). As brain T2‐hyperintense lesions also appear in other conditions, this can contribute to misdiagnosis (Kaisey et al., 2019; Solomon, Bourdette, et al., 2016). Additional biomarkers have been used and suggested to aid in improving the confirmation of clinically definite MS.

The central vein sign (CVS) has been proposed to discriminate MS lesions from MRI mimics such as microvascular disease and migraine by identifying a blood vessel in the center of a T2 hyperintense lesion (Sati et al., 2016; Tallantyre et al., 2011). Several studies have evaluated the incidence, sensitivity, and specificity of the CVS in distinguishing MS versus non‐MS lesions across different MRI sequences that detect field inhomogeneities including T2‐weighted, susceptibility‐weighted, and phase imaging (Clarke et al., 2019; Dworkin et al., 2018; Maggi et al., 2019; Mistry et al., 2015; Sinnecker et al., 2019). While specific percentages of lesions containing CVS vary by brain region, they occur significantly more often than other neurological or cerebrovascular pathologies (Kilsdonk et al., 2014; Sati et al., 2016; Solomon, Schindler, et al., 2016; Wuerfel et al., 2012). Across these studies, CVS had a specificity for MS by approximately 80% or higher, prompting ongoing research to determine the consistency of CVS in MS across a large, multicenter population (Ontaneda, Sati, et al., 2021).

Oligoclonal immunoglobulin bands (OCBs) in the cerebrospinal fluid (CSF) are included in the most recent revision of the McDonald criteria to aid in diagnosis of MS by detecting B cell activation (Obermeier et al., 2011; Thompson et al., 2018). Unique OCBs present in the CSF and not present in the serum (both obtained from a patient on the same day), suggests intrathecal production of immunoglobulins directed against ubiquitous intracellular antigens in cellular debris (Winger & Zamvil, 2016), corroborating the presence of CNS inflammation. Patients diagnosed with CIS who have OCBs have a greater risk of “conversion” to multiple sclerosis (Dobson et al., 2013). OCBs are not specific to MS as they can be found in over 30 conditions (Chu et al., 1983; Petzold, 2013), but overall have predictive value in combination with MRI when the history and exam make the diagnosis of MS difficult to ascertain. These bands can also, therefore, clarify a case of clinically definite MS when MRI alone cannot determine a diagnosis.

#### 2.1.2 Evaluating disease activity

Following a diagnosis of MS, there is a critical need to monitor new disease activity and assess response to treatments. MRI sequences imaging T2‐weighted, fluid‐attenuated inversion recovery (FLAIR), and T1‐weighted with or without gadolinium enhancement have been used both to diagnose MS and to monitor evidence of disease activity (Trip & Miller, 2005; Wattjes et al., 2015). In particular, gadolinium enhancement represents breakdown of the blood–brain barrier and correlates with inflammation (Grossman et al., 1986). The FLAIR sequence suppresses signal from the CSF, which is hyperintense in T2‐weighted imaging, allowing for better detection of periventricular lesions (Barkhof & Scheltens, 2002; Trip & Miller, 2005). T2‐lesion counts and volume are also practical outcome measures of treatment efficacy in clinical trials (Miller et al., 1996; Polman et al., 2003, 2006; van Munster & Uitdehaag, 2017). As MS pathology is heterogeneous and MRI may not always capture definitive disease‐specific activity, additional methods to detect inflammatory activity in addition to MRI have been proposed to aid in monitoring clinical changes.

The B cell chemoattractant CXCL13 in CSF has been associated with the conversion from CIS to definitive MS (Khademi et al., 2011). Additionally, serum and CSF levels of this molecule have been associated with disease activity, number of relapses, and Expanded Disablity Status Scale (EDSS) score, suggesting its potential as a marker of inflammatory exacerbations in MS (DiSano et al., 2020; Festa et al., 2009; Khademi et al., 2011). Neurofilament light chain (NfL) in the serum (sNfL) or CSF is also indicative of neuronal or axonal damage, as neurofilaments are essential scaffolding proteins of the axon cytoskeleton that are shed during damage (Petzold, 2005; Yuan et al., 2017). While not a specific feature to MS (and therefore not used for the purposes of diagnosis), sNfL and CSF NfL have been shown to be elevated throughout MS disease courses and are associated with EDSS score (Disanto et al., 2017; Kuhle et al., 2013; Malmeström et al., 2003). sNfL in particular is less invasive to sample than the CSF, and has been correlated with lesion activity, relapses, and axonal damage (van den Bosch et al., 2022; Yik et al., 2022). sNfL levels are affected by active disease, making its utility as a predictor of transition from relapsing to progressive disease more difficult (Kapoor et al., 2020). Despite this, sNfL is proposed to have predictive value for disease prognosis and treatment response (Kapoor et al., 2020; Thebault et al., 2021).

#### 2.1.3 Monitoring neurodegeneration

In addition to accurately diagnosing and treating inflammatory disease activity in MS, there is a need for markers that can predict and detect progressive decline and permanent disability caused by neurodegeneration. MRI quantification of CNS atrophy is one such indicator. Brain atrophy in various regions of the gray and white matter occurs early in the course of MS and has been associated with cognitive dysfunction and overall worsening of clinical disability (Chard et al., 2002; Houtchens et al., 2007; Jacobsen et al., 2014). Cortical thinning and deep gray matter atrophy in structures such as the caudate, putamen, thalamus, and hippocampus are some of the earliest predictors of disease progression and can be detected even during CIS (Calabrese et al., 2007; Eshaghi et al., 2018; Ontaneda, Raza, et al., 2021). Thalamic atrophy observed by MRI occurs early in the disease course and is one of the strongest correlates of disease progression (Azevedo et al., 2018; Hänninen et al., 2019; Štecková et al., 2014; Zivadinov et al., 2013). Cervical spinal cord atrophy is also an important indicator of disease progression, predicting conversion to SPMS even in the absence of clinical relapses or other disease activity (Bischof et al., 2021; Lin et al., 2003). Additionally, a large retrospective study by Genovese et al. (2019) showed that T2‐lesions replaced by CSF (termed “atrophied T2 lesion volume”) increase the probability of conversion to SPMS. Therefore, these measures of neurodegeneration and disease progression may provide important insight for clinical interventions and future therapies to treat progressive disease.

The visual system also serves as a non‐invasive indicator of neurodegeneration in MS. Optical coherence tomography (OCT) imaging of the retina measures the structural integrity and thicknesses of its distinct cellular layers (Frohman et al., 2006; Huang et al., 1991). Retinal thinning has been correlated with clinical disability and brain atrophy in MS independent of optic neuritis (Albrecht et al., 2012; Gordon‐Lipkin et al., 2007; Saidha et al., 2015), suggesting a utility in evaluating neurodegeneration. Greater rate of degeneration of the inner and outer nuclear layers in patients with progressive MS independent of age (Sotirchos et al., 2020), and association with clinical disability (Saidha et al., 2012) further support exploring the visual pathway to recognize neurodegenerative changes early in the MS disease course. Measures of neuronal function have also provided a way to assess extent of demyelination in MS. Visual evoked potentials (VEP) measure neuronal responses to a visual stimulus (Odom et al., 2016). In MS, demyelination in the visual system including the optic nerve, optic radiation, or lateral geniculate nucleus of the thalamus are common and reflected as a delay in VEP response time (Barton et al., 2019; Poser et al., 1983). Recovery of nerve function can also be assessed with this measure (Barton et al., 2019; Halliday & McDonald, 1977; Hickman et al., 2004). Delayed VEPs are associated with higher EDSS score, suggesting that they may also have predictive value for worsening disease (Fuhr et al., 2001).

Serum levels of glial fibrillary acidic protein (GFAP) are elevated in progressive MS patients and associated with disease duration and clinical disability (Högel et al., 2020). GFAP levels correlate with NfL, lesion load, and brain volume for both white and gray matter in progressive MS, suggesting that GFAP may be an indicator of disease progression (Axelsson et al., 2011; Ayrignac et al., 2020; Högel et al., 2020). It should be noted that the strength of correlation between GFAP and disease progression is not ascertained, as earlier studies suggest a mild to moderate relationship between serum GFAP and increased disability (Martínez et al., 2015; Norgren et al., 2004).

MRI remains the standard of care for detecting, diagnosing, and monitoring MS inflammatory activity and neurodegeneration (Thompson et al., 2018). While EDSS and relapse rate are often primary outcome measures in clinical trials, evidence of disease activity by MRI is also a primary or secondary outcome measure to determine the efficacy of pharmacologic therapies (van Munster & Uitdehaag, 2017). Each outcome measure has its own limitations, but these studies suggest that determining clinically definite MS and detecting neurodegenerative changes may occur more accurately and more quickly in combination with serum and CSF biomarkers, which may in turn improve overall quality of care.

### 2.2 Disease courses

**FIGURE 1:**
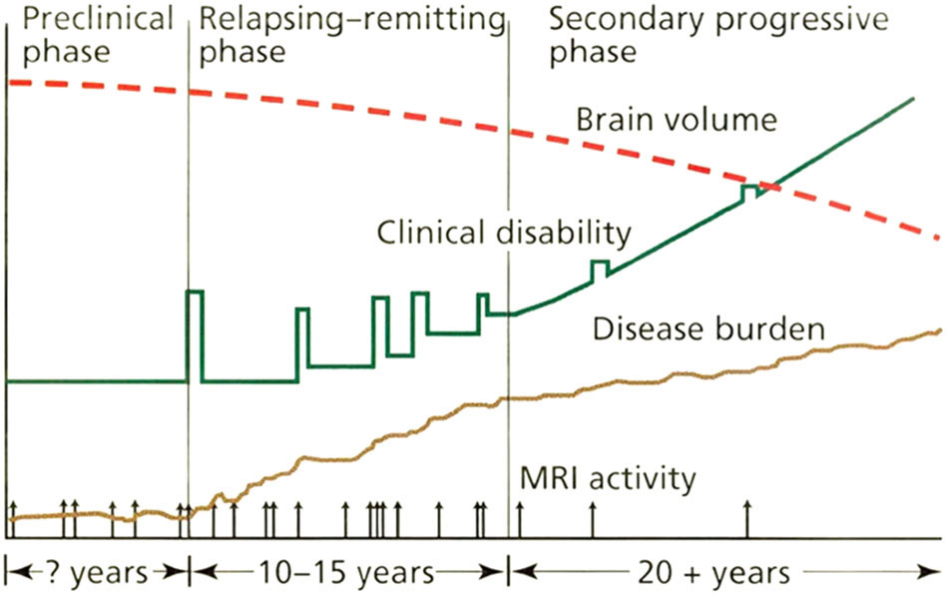
Major multiple sclerosis disease courses. MS is diagnosed following a preclinical phase of an unknown period of time in which inflammatory activity may occur (black arrows), but does not reach clinical threshold. Inflammatory lesions as detected by MRI that cause clinical symptoms which resolve over time (green line) contribute to the relapsing–remitting disease course. However, despite remission of clinical symptoms, subclinical inflammatory activity may still occur as seen upon MRI and contributes to accumulating disease burden and loss of brain volume (brown line and red dashed line, respectively). Eventually, the disease can reach a secondary progressive course which is characterized by increasing disability and brain atrophy despite fewer inflammatory events. This phase of disease is an important area of research, as physiological mechanisms of remyelination and axon repair cannot overcome the damage that ultimately drives disease progression in MS. Reprinted with permission from Fox and Cohen (2001). Copyright © 2001. Cleveland Clinic Foundation. All rights reserved.

According to the 2017 revision of the McDonald criteria, MS is diagnosed by the presence of T2‐weighted hyperintense lesions that are disseminated in space and time, which may include the presence of OCBs in the CSF (Thompson et al., 2018). Patients with MS can experience a range of symptoms and phenotypes with or without inflammatory disease activity in different clinical courses (Figure 1). The most common disease course is RRMS, which accounts for nearly 85% of all cases (Confavreux et al., 2000). This is characterized by periods of clinical disability, often accompanied by active CNS inflammation, followed by variable recovery (Sotiropoulos et al., 2021; Trapp & Nave, 2008). A wide range of symptoms include motor deficits, fatigue, pain, and visual impairments. Although a patient may clinically be in remission, MRI disease activity is evident (Figure 1) suggesting that lesions contributing to secondary neurodegeneration are occurring during the relapsing–remitting phase of MS. This is also consistent with brain atrophy occurring early in disease that can worsen with or without inflammatory activity across all disease courses. Some patients who initially had a relapsing course can later develop gradual disability worsening over time (SPMS). Studies noting a high proportion of RRMS patients who will later develop SPMS were diagnosed with MS in an era with low efficacy DMTs. The early use of high efficacy DMTs (i.e., more effectively reducing relapse rate), is predicted to prevent accumulation of lesions, subsequent neurodegeneration, and both delay the time to developing SPMS and mitigate the rate of clinical decline (Buron et al., 2020; Filippi et al., 2022). Table 1 describes current DMTs and their mechanisms of action to mitigate inflammatory relapses. A smaller proportion (~15%) of people with MS have PPMS, with gradually increasing disability from the onset of disease. Both SPMS and PPMS can include inflammatory activity, necessitating routine clinical and periodic MRI surveillance (Lublin et al., 2014). While inflammatory events as observed by MRI are essential to the diagnosis of MS and monitoring disease activity, a clinic–radiographic paradox exists in the modest correlations of disability with global T2‐lesion burden in progressive MS (Koch et al., 2021; Korteweg et al., 2008; Li et al., 2006; Mostert et al., 2010). Possible mechanisms include “critical” lesions impacting clinically eloquent tracts (e.g., corticospinal) in the brainstem and spinal cord impacting ambulation and secondary neurodegenerative changes (e.g., neuroaxonal loss and mitochondrial dysfunction) occurring as a sequela to prior lesions.

**TABLE 1:** Disease‐modifying therapies for the treatment of MS

DMT	Proposed mechanisms of action	References
*High efficacy*		
Natalizumab	Humanized monoclonal antibody binding to α_4_β_1_‐integrin; prevents leukocyte infiltration into CNS	Miller et al. (2007); Yednock et al. (1992)
Alemtuzumab	Humanized monoclonal antibody binding to CD52 on T and B lymphocytes; depletes from circulation	Cohen et al. (2012); Hu et al. (2009)
Ocrelizumab, ofatumumab, rituximab	Humanized, human, or chimeric monoclonal antibodies binding to CD20 on B lymphocytes; depletes from circulation	Chisari et al. (2022); Hauser et al. (2016); Hauser et al. (2020); Montalvao et al. (2013)
*Moderate efficacy*		
Fingolimod, siponimod, ozanimod, posanimod	Structural analog to sphingosine; competitive inhibitor for sphingosine‐1‐phosphate; reduces lymphocyte egress into circulation	Behrangi et al. (2019); Calabresi et al. (2014); McGinley and Cohen (2021); Mehling et al. (2008); Pinschewer et al. (2000)
Cladribine	Structural analog to adenosine; competitive inhibitor for adenosine deaminase; disrupts DNA synthesis and repair, leads to preferential T and B lymphocyte depletion	Carson et al. (1983); Giovannoni et al. (2010); Leist et al. (2014); Seto et al. (1985)
Dimethyl fumarate, diroximel fumarate	Promotes anti‐oxidant activity in CNS and periphery by Nrf2 activation; reduces overall T and B cell numbers in circulation; reduces lymphocyte maturation and activation	Fox et al. (2012); Gold et al. (2012); Mills et al. (2018); Spencer et al. (2015)
*Low efficacy*		
Interferon beta‐1a and interferon‐1b	Recombinant human interferon‐beta; increases anti‐inflammatory and decreases proinflammatory signaling by peripheral immune cells	Dubois et al. (2003); Filipi & Jack (2020); Kieseier (2011)
Glatiramer acetate	Competes with MHC and TCR binding, thereby reducing inflammatory signaling; reduces proinflammatory signaling by inducing a shift toward T regulatory cell expression	Boster et al. (2011); Johnson et al. (1995); Neuhaus et al. (2001)
Teriflunomide	Reversible inhibitor for dihydro‐orotate dehydrogenase; preferentially inhibits lymphocyte proliferation leading to overall reduction of activated T and B cells	Bar‐Or et al. (2014); O'Connor et al. (2011)

Abbreviations: MHC, major histocompatibility complex; Nrf2, nuclear factor erythroid 2 related factor 2; TCR, T cell receptor.

### 2.3 Inflammatory lesion heterogeneity in white matter

It is well‐established that MS lesions have differing phenotypes based largely on the status of immune cell infiltration, glial activation, and demyelination (Bö et al., 1994; Frischer et al., 2015; Lucchinetti et al., 1996, 2000; Traugott et al., 1983; van Horssen et al., 2012). These inflammatory characteristics are also associated with axonal loss (Ferguson et al., 1997; Trapp et al., 1998) and have been used to categorize lesion patterns as active, chronic active, or chronic inactive (Bö et al., 1994; Kuhlmann et al., 2017).

Active lesions are characterized by hypercellularity, containing vast infiltrates of macrophages, T cells, and B cells often in perivascular cuffs and in the center of demyelinated regions (Bö et al., 1994; Frischer et al., 2009; Machado‐Santos et al., 2018; Traugott et al., 1983). Macrophages and microglia contain engulfed fragments of minor and major myelin proteins, such as myelin‐associated glycoprotein (MAG) and proteolipoprotein (PLP), respectively (Lucchinetti et al., 1996; Prineas et al., 1984). This demyelinating activity and pattern of inflammation in active or early demyelinating lesions can be further broken down into four patterns as defined by Lucchinetti et al. (2000). Pattern I and II lesions were shown to be fairly similar, with inflammation characterized primarily by T cell and macrophage infiltrates. Pattern II lesions in particular, which were the most commonly found lesion type, exhibited significant immunoglobulin (Ig) and complement deposition in areas of myelin degradation, which was not observed in Pattern I lesions (Lucchinetti et al., 2000). Demyelinated regions are centered near veins and venules, highlighting the role of infiltrated immune cells through a penetrable blood–brain barrier in lesion pathology (Barnett & Prineas, 2004; Gaitán et al., 2011). Pattern III lesions, in contrast, are devoid of Ig and complement deposition, though still have inflammatory activity characterized by T cell and macrophage infiltration with microglial activation. Pattern III lesion borders are not well defined, with rims of myelin preserved around inflamed vessels within the demyelinated area and evidence of oligodendrocyte degradation and apoptosis (Lucchinetti et al., 2000; Zrzavy et al., 2017). Finally, rare Pattern IV lesions, similar to Pattern III, contain T cells and macrophages and do not have Ig or complement deposition. Demyelinated areas are associated with dying oligodendrocytes as shown with markers of DNA fragmentation, but morphological characteristics of apoptosis that were observed in Pattern III lesions are absent (Lucchinetti et al., 2000). These heterogeneous active lesions are found primarily in RRMS and early in disease course, though some are also found in progressive forms of MS (Frischer et al., 2015). The four patterns of active lesions highlight the varying histopathological changes that occur in MS as observed in *post‐mortem* brain tissue. These active lesions are found in all disease courses (with the exception of the rare Pattern IV, which was found only in chronic disease) (Lucchinetti et al., 2000).

**FIGURE 2:**
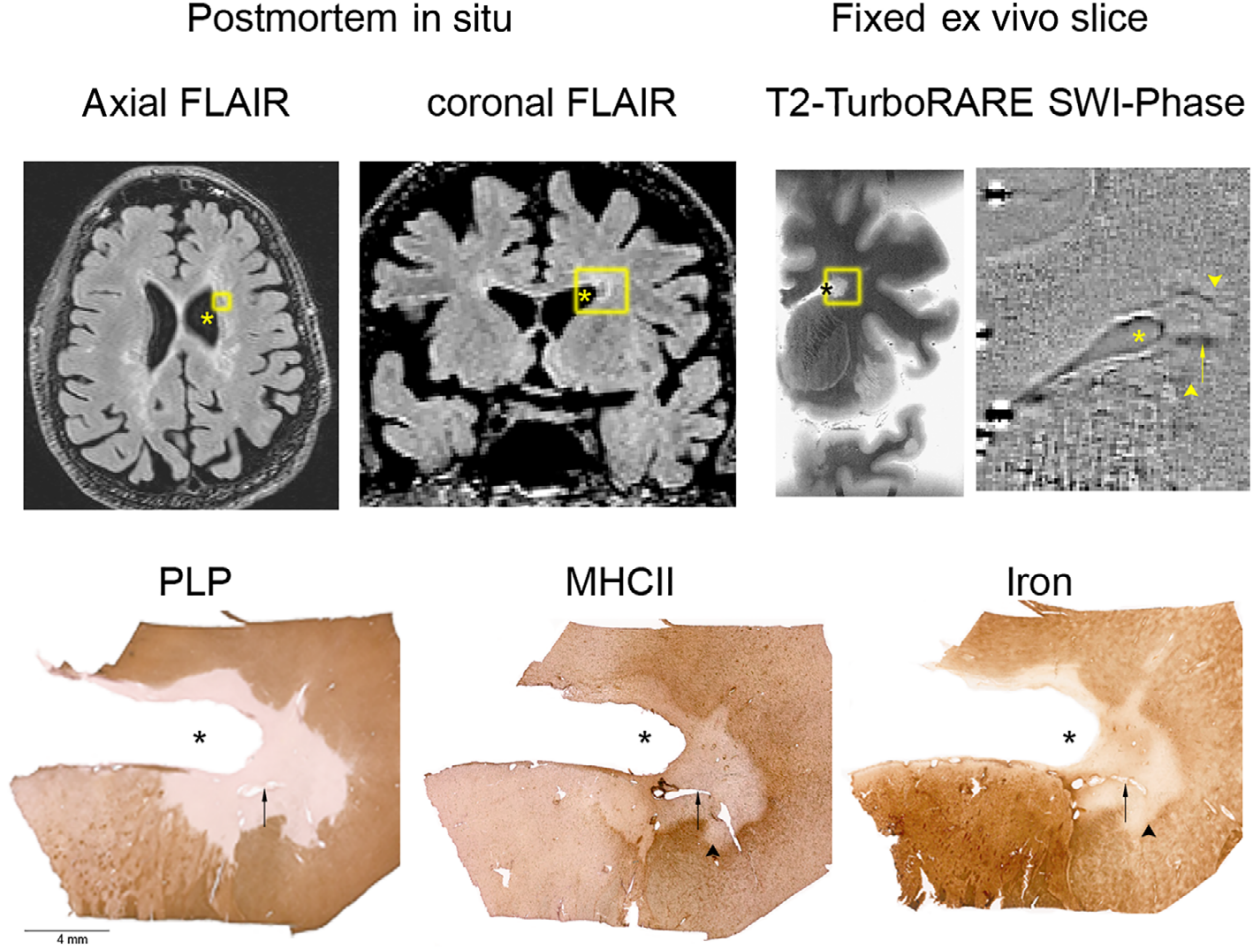
Lesion pathology in multiple sclerosis. MRI‐pathology correlations of a periventricular lesion with a paramagnetic rim and vessel. FLAIR images obtained in a post‐mortem in situ 3 T MRI (axial and coronal planes shown) demonstrate a periventricular T2‐hyperintense lesion outlined in yellow with the lateral ventricle noted with an asterisk. A 7 T MRI with a T2‐weighted image shown was done following fixation of the 1‐cm coronal tissue slice with the lesion outlined. The lesion border has a paramagnetic rim and a vessel in its center on SWI‐phase imaging. Immunohistochemistry of 30‐μ free floating sections of the area was stained for myelin (PLP) and activated microglia/macrophages (MHCII) and demonstrates a demyelinated periventricular chronic active lesion. A modified Turnbull iron histological stain shows areas of increased iron at the lesion border coinciding with MHCII and the SWI‐phase RIM (arrowhead). FLAIR, fluid‐attenuated inversion recovery; MHCII, major histocompatibility complex II (activated microglia/macrophages); PLP, proteolipid protein (myelin); SWI, susceptibility weighted imaging; T, Tesla; T2‐Turbo‐RARE, T2‐weighted Turbo Rapid Imaging with Refocused Echoes.

Chronic active lesions are characterized by hypocellular demyelinated centers containing reactive astrocytes and hypercellular rims (Bö et al., 1994; Kuhlmann et al., 2017), a subset of which contain iron‐rich activated microglia and macrophages. On MRI, some lesions may contain a paramagnetic rim that has been associated with slow expansion of the lesion, also referred to as “smoldering” (Calvi et al., 2020; Frischer et al., 2015). Figure 2 demonstrates a post‐mortem MRI‐pathology correlation of a periventricular lesion that was slowly expanding while the subject was living, as well as a paramagnetic rim, and a central vein sign. Histopathological analysis showed a chronic active lesion with loss of myelin (PLP) and increased microglia/macrophages (MHCII) at the lesion border with few MHCII‐expressing macrophages enriched in iron, consistent with findings that slowly expanding lesions with rims are histologically associated with iron‐containing microglia (Dal‐Bianco et al., 2017). Microglia/macrophages at the rim of chronic active lesions in *post‐mortem* tissue contain myelin degradation products, whereas smoldering lesions tend to have less demyelinating activity (Frischer et al., 2015; Popescu & Lucchinetti, 2012). Smoldering lesions are also observed more frequently in progressive MS and therefore may be indicative of slowed demyelination with an increase in axonal and neuronal degeneration (Frischer et al., 2009, 2015). The inflammatory rim of chronic active lesions has been assessed for cellular interactions that may contribute to neurodegeneration. Absinta et al. (2021) identified through single‐nucleus RNA sequencing from *post‐mortem* MS brains that activated microglia and astrocytes associated with chronic active lesions had neurodegenerative transcriptional profiles. In particular, the complement activation component C1q has been identified in areas of active demyelination (Breij et al., 2008) and its transcript was found to be upregulated in the activated astrocytes and microglia associated with the rims of chronic active lesions (Absinta et al., 2021). These studies aid in the understanding of how neurodegeneration may occur in lesions with inflammatory activity.

Inactive lesions, on the contrary, are hypocellular and any present macrophages or microglia contain little to no myelin degradation products (Bö et al., 1994; Kuhlmann et al., 2017). Inactive lesions have a clear border and are observed predominantly in progressive MS (Frischer et al., 2015). Although these lesions do not exhibit inflammatory activity, over time newly infiltrating immune cells induce inflammatory activity that has been observed by MRI (Campbell et al., 2012; Kuhlmann et al., 2017; Thompson et al., 1991). Aside from reactivation of inflammatory activity, some lesions will also remyelinate. The potential for remyelination is typically found earlier in the disease course, as oligodendrocyte progenitor cells (cells that mature to form the myelin sheath) appear within active lesions as opposed to chronic lesions (Barkhof et al., 2003; Frischer et al., 2015; Patrikios et al., 2006). Additionally, those with progressive MS are less likely to experience remyelinated lesions, also referred to as “shadow plaques,” than those with relapsing MS (Frischer et al., 2015; Goldschmidt et al., 2009; Patrikios et al., 2006; Prineas et al., 1993). The failure of lesions to remyelinate is still not completely understood, and speaks to the importance of better understanding the mechanisms underlying MS pathogenesis and disease progression. In addition, while much of the early research in MS focused on white matter pathology, gray matter lesions and pathology provide important insight into the disease complexity and heterogeneity.

### 2.4 Cortical lesions and gray matter pathology

Evidence of cortical lesions are observed in both relapsing and progressive forms of MS, with lesions in the cortex or in juxtacortical positions representing approximately 20%–25% of all lesions and appearing in up to 90% of MS cases based on *post‐mortem* analyses (Albert et al., 2007; Brownell & Hughes, 1962; Geurts et al., 2005; Junker et al., 2020; Kidd et al., 1999; Kutzelnigg et al., 2005). Seven types of cortical lesions have been defined based on their localization in the brain (Kidd et al., 1999), which have been simplified to three predominant categories (Bø et al., 2003; Junker et al., 2020; Peterson et al., 2001). Type I (leukocortical or juxtacortical) are the most prevalent type of cortical lesions and begin in the subcortical white matter, but extend into the cortex and remain below the surface of the brain. Type II (intracortical) lesions lie within the cortex and do not extend to the cortical surface or subcortical white matter. These lesions are typically perivascular (Junker et al., 2020; Trapp & Nave, 2008). Type III lesions are characterized by extensive subpial demyelination presenting either as ribbons across multiple gyri or wedges starting at the brain surface. This type of lesion has been shown to be specific in MS compared with other demyelinating diseases (Junker et al., 2020). Juxtacortical and intracortical lesions are perivenular and mediated by the infiltration of peripheral immune cells, while subpial lesions are likely the result of demyelination by a CSF factor not yet identified (Junker et al., 2020). A fourth type of cortical lesion has also been classified with extensive demyelination across the entire cortex, but with no extension into the subpial white matter (Bø et al., 2003). While not as common, this type of cortical demyelination highlights the extent to which the cortex can be affected without significant evidence of disease activity in the subpial white matter. Furthermore, *post‐mortem* analysis of a subset of MS brains has been categorized as myelocortical MS. This subgroup is defined as exhibiting demyelinated lesions in the cortex and spinal cord without significant demyelination in the subcortical white matter despite subcortical MRI T2‐hyperintensities (Trapp et al., 2018). Characterization of gray matter pathology in MS has provided insight into the multifactorial inflammatory and degenerative processes that contribute to clinical disability.

Cortical lesions differ from white matter lesions, typically having fewer infiltrating and resident immune cells as well as an intact blood–brain barrier (Junker et al., 2020; Kidd et al., 1999; Peterson et al., 2001). Demyelinated cortical regions exhibit transected neurites and apoptotic neurons that correlate with inflammatory activity (Peterson et al., 2001), and greater cortical lesion burden is associated with cognitive impairment and clinical disability progression in MS (Calabrese et al., 2009; Calabrese et al., 2012; Forslin et al., 2018; Harrison et al., 2015). Although cortical lesions may have fewer infiltrating immune cells, ectopic B‐cell follicle‐like structures have been observed in the meninges of MS tissue near subpial lesions (Howell et al., 2011; Magliozzi et al., 2007). This suggests a pathological humoral response that may contribute to cortical pathology. Additionally, while the inflammatory infiltrates may be fewer relative to white matter lesions, microarray analyses have shown that T cell‐mediated inflammation, microglial activation, and oxidative stress are characteristics of cortical lesions that lead to demyelination and axonal loss (Fischer et al., 2013). Crosstalk between peripheral immune cells and resident microglia or astrocytes may contribute to meningeal inflammation and cortical lesion formation, contributing to the varying cortical lesion types (Zuroff et al., 2021).

The caveat to cortical lesion burden as an indicator of MS progression is their obscure identification by MRI. Thus, cortical lesion analysis has been performed primarily *post‐mortem* (Geurts et al., 2005; Kidd et al., 1999). Focal lesions appear across all MS disease courses in many deep gray matter regions such as the hypothalamus, caudate, putamen, amygdala (Haider et al., 2014; Vercellino et al., 2009), and thalamus (Mahajan et al., 2020; Ontaneda, Raza, et al., 2021). Histopathology and MRI of the thalamus in *post‐mortem* MS brains support that thalamic volume correlates better with damage in extra‐thalamic regions than with lesions directly in the thalamus (Mahajan et al., 2020). This suggests that gray matter atrophy may result from loss of efferent and afferent axonal pathways in white matter tracts. As axonal loss is a major component of disability accumulation in MS, identifying atrophied regions that may predict disease progression will help identify patients with MS who are more at risk for neurodegenerative changes.

### 2.5 Axonal loss and atrophy

Axonal transection and eventual degeneration are known to occur within demyelinated lesions and correlate with clinical disability (Trapp et al., 1998). In addition, Wallerian degeneration early in the disease course contributes to loss of axons following demyelination of their distal counterparts (Dziedzic et al., 2010; Singh et al., 2017; Box 1).

BOX 1 **Thalamic and spinal cord atrophy are early indicators of MS disease progression**

As depicted in Figure 1, the appearance of new lesions by MRI can occur even during a clinical remission (Fox & Cohen, 2001). These “subclinical” lesions suggest ongoing CNS inflammation despite a partially recovered or non‐progressing neurological status. Lesions presenting in eloquent locations may be fewer in number, but present greater risk for disability and progressive decline (Krieger et al., 2016; Sechi et al., 2019). Monitoring demyelinating lesions is important to track disease activity, treatment response, and risk for progression, but their variable development and clinical manifestation limit their use as an early predictor of disease worsening. Sustained inflammation and demyelination throughout MS contributes to axonal loss and neurodegeneration, leading to CNS atrophy. Thalamic and spinal cord atrophy have been identified as independent predictors for greater clinical disability and shorter time to disease progression (Azevedo et al., 2018; Bischof et al., 2021; Lin et al., 2003; Štecková et al., 2014). Thalamic and spinal cord atrophy are apparent across all MS disease courses (Azevedo et al., 2018; Bernitsas et al., 2015), making them useful as early imaging markers of neurodegenerative changes. Assessing thalamic and spinal cord atrophy also accounts for neurodegeneration that may occur independently of damage from focal demyelinating lesions, and captures damage from subclinical lesions that contribute to disease progression. MRI biomarkers for CNS atrophy early in disease course will help determine disease prognosis on an individual basis and highlight the need for neuroprotective therapies very early in disease.

Throughout MS there is a potential for worsening of disease without overt disease activity in the form of new/enlarging lesions or clinical relapses. This “silent progression” is insidious and could be explained by injury to clinically eloquent regions such as the corticospinal tract by (1) secondary neuroaxonal loss as a sequela to prior disease activity; (2) disease activity affecting the regions infrequently imaged (e.g., spinal cord); and (3) limited imaging resolution or obscuration by artifact. Neurodegenerative changes can be visualized by cortical thinning, whole and regional (e.g., thalamic) brain atrophy, and spinal cord atrophy (e.g., upper cervical cord cross sectional area). As mentioned, thalamic atrophy measured by MRI and neuronal loss in *post‐mortem* tissue have also been observed in MS independent of focal lesions (Cifelli et al., 2002; Mahajan et al., 2020). Additionally, thalamic atrophy has been shown to occur early in MS disease course at a rate significantly faster than healthy controls in a longitudinal study of more than 500 subjects with RRMS (Azevedo et al., 2018). Bischof et al. (2021) showed that cervical spinal cord atrophy in particular predicts conversion to SPMS, as spinal cord atrophy rates were increased up to 4 years prior to conversion from RRMS to SPMS. Furthermore, a 1% faster rate of spinal cord atrophy was associated with more than 50% shorter time to silent progression and conversion to SPMS (Bischof et al., 2021). A decreased cervical spinal cord area has also been associated with increased disability over time, independent of spinal cord lesion load (Losseff, Webb, et al., 1996; Weeda et al., 2022). Of note, spinal cord lesions may also serve as risk factors for increasing CNS atrophy in progressive MS. Lesions in critical locations within the corticospinal tract such as the lateral spinal cord have been associated with progressive motor impairment, attributed to few or even a single lesion (Keegan et al., 2016; Sechi et al., 2019).Together these data emphasize that neuro‐axonal loss in the brain and spinal cord are associated with an increased risk of disease worsening, and that thalamic and spinal cord atrophy may provide useful imaging correlates to identify MS progression.

Despite these advances in predicting disease progression, the underlying mechanisms driving neurodegeneration must be elucidated in order to develop neuroprotective strategies. Animal models of demyelinating disease have provided useful insight into the relationship between demyelination, axonal injury, and neurodegeneration and how to promote remyelination and axonal repair.

## 3 MECHANISMS OF NEURODEGENERATION IN MS

Myelinated axons require proper support from oligodendrocytes in order to cluster sodium channels for saltatory conduction which increases signal transmission speed (Kaplan et al., 1997), reduces axonal damage during environmental insult (Pitt et al., 2010), and maintains metabolic function (Fünfschilling et al., 2012; Lee et al., 2012). During MS and animal models of demyelinating disease, loss of oligodendrocytes and the myelin sheath therefore impairs proper axonal function and metabolism. As axonal loss is a key contributing factor to progressive neurological disability in MS (Trapp & Nave, 2008), protecting the integrity of the axon is an important area of research. Data from animal models of demyelinating disease have provided important insight into factors leading to neurodegeneration and potential mechanisms of neuroprotection including remyelination and/or restoration of neuronal function.

While no animal models perfectly recapitulate all aspects of MS, there are several models available to study inflammatory, demyelinating, and neurodegenerative events to better understand MS pathology so that improved therapeutic strategies can be developed. The most commonly used model is experimental autoimmune encephalomyelitis (EAE), which is an adaptive immune‐mediated model whereby CNS–infiltrating myelin–reactive T cells initiate autoimmune demyelination and axon loss (t Hart et al., 2021; Wujek et al., 2002). When actively induced with myelin oligodendrocyte glycoprotein (MOG) peptides in C57BL/6J mice, EAE results in an ascending paralysis due to demyelinating lesions primarily in the spinal cord (Mendel et al., 1995). This is followed by a chronic phase of disease representing sustained demyelination after peak immune cell infiltration (Tompkins et al., 2002). Alternatively, EAE can be induced using a proteolipoprotein (PLP) peptide in SJL/J mice, which similarly induces a T cell‐mediated autoimmune response to myelin and ascending paralysis, but is accompanied by pathology in the brain and a relapsing–remitting clinical phenotype (McRae et al., 1992). EAE has also been induced in Lewis rats, which presents as acute paralysis with spontaneous recovery (Swanborg, 2001), or in marmosets, which allows the investigation of demyelinating disease in nonhuman primates (Brok et al., 2001) and may provide important therapeutic insight (Stassart et al., 2016). Another method of inducing demyelination is through viral injection. For the purposes of studying MS, Theiler's murine encephalomyelitis virus (TMEV) is a common viral method to study the effects of inflammatory demyelination, remyelination, and axonal loss (Clatch et al., 1987; McGavern et al., 1999; Oleszak et al., 2004; Tsunoda et al., 2003).

There are also models that allow the investigation of demyelination and axonal loss without robust inflammatory responses to help delineate the mechanisms underlying demyelination and remyelination. These models utilize chemical‐ or toxin‐induced demyelination that are followed by remyelination, a recovery that may be observed in certain EAE and viral models only partially or not at all. Two important models, though there are others, are cuprizone and lysolecithin‐induced demyelination. Cuprizone is a copper chelator that, when ingested in chow over several weeks, induces vast demyelination often studied in the corpus callosum. When a normal chow diet resumes, successful remyelination can then be studied in the absence of an adaptive immune response (Lindner et al., 2008; Matsushima & Morell, 2001). Similarly, lysolecithin‐induced demyelination is followed by remyelination. This toxin cleaves lipids and is injected to induce focal demyelinating lesions (Pavelko et al., 1998; Plemel et al., 2018). In this respect, remyelinating processes can be investigated relative to normal‐appearing tissue. Overall these models provide insight into pathological and reparative mechanisms that occur, or in the case of remyelination, fail, in MS. Together studies utilizing these models can help elucidate targetable mechanisms of degeneration in MS to promote recovery and repair.

### 3.1 Impaired axonal transport and mitochondrial dysfunction

As mentioned, the EAE model in C57BL/6J mice is one of the most commonly used models to test therapeutics for the treatment of MS (t Hart et al., 2021). In the EAE model, mitochondrial dysfunction in axons is a consequence of demyelination (Nikić et al., 2011) before overt axonal pathology is apparent. Mitochondrial trafficking is an essential component of neuronal mitostasis: the mechanism for maintaining a healthy, functioning pool of mitochondria necessary for the overall health of an axon (Misgeld & Schwarz, 2017). Additionally, in some axons aberrant accumulation of mitochondria may occur before overt demyelination or structural changes to the axon, which may be caused by reactive oxygen species generated during acute inflammation (Sorbara et al., 2014). Mitochondrial trafficking has also been shown to be disrupted within inflammatory lesions following damage to the axon, suggesting that this phenomenon is both an early and persisting contributor to axonal dysfunction in EAE (Nikić et al., 2011; Sorbara et al., 2014). Impairments in axonal mitochondrial trafficking are also apparent in the lysolecithin‐induced model of demyelination, a toxin‐induced method of myelin loss that is not initiated by a peripheral inflammatory response (Licht‐Mayer et al., 2020). These studies also demonstrated that enhancing neuronal mitochondrial biogenesis and transport reduced axonal degeneration, suggesting that proper mitochondrial transport is essential for axonal recovery and protection during neuroinflammation and demyelination (Licht‐Mayer et al., 2020). Respiratory chain deficiencies, alterations in mitochondrial content, and mitochondrial DNA mutations have been also observed in *post‐mortem* cortical (Dutta et al., 2006) and spinal cord (Licht‐Mayer et al., 2020) specimens from progressive MS. This indicates that there are persistent alterations in mitochondrial function contributing to progressive axonal degeneration. These studies highlight the importance of early detection of MS to optimize therapeutic strategies that protect the axon following inevitable demyelinating events.

### 3.2 Chronic demyelination and neurodegeneration

Loss of synaptic proteins has been observed following demyelination in *post‐mortem* MS brain specimens and in the EAE model (Dutta et al., 2011; Werneburg et al., 2020). Demyelination and axonal injury is thought to trigger a complement‐mediated microglial response that serves to clear dysfunctional synapses, damaged axons, and stressed/dying neurons (Michailidou et al., 2015; Watkins et al., 2016; Werneburg et al., 2020). Synaptic loss has been observed in the hippocampus during EAE, along with hippocampal atrophy and diffuse demyelination in the absence of infiltrating T cells (Ziehn et al., 2010). In MS, hippocampal atrophy particularly of the CA1 region has been observed and correlates with worsened performance on memory tasks (Sicotte et al., 2008). Synaptic loss by immunofluorescence and electron microscopy has also been observed in the dorsal lateral geniculate nucleus (LGN) of the thalamus in EAE (Mey et al., 2022; Werneburg et al., 2020), and we recently showed that thalamic volume in MS (and specifically the LGN) correlates with neuroperformance (Mey et al., 2022). This suggests that in the EAE model, like in MS, neurodegeneration can occur outside of regions directly exposed to focal demyelinating lesions. Increased GABA‐mediated tonic inhibition is observed in the hippocampus during EAE, which reduces hippocampal excitability and long‐term potentiation (Kammel et al., 2018). Loss of synapses and altered synaptic transmission have implications for cognitive decline in learning and memory (Ziehn et al., 2010), with important relevance to MS as cognitive changes are evident in both relapsing–remitting and progressive phases of disease (Ruano et al., 2017).

In the cuprizone model of demyelination, reduced synaptic transmission has been observed in the hippocampus and visual cortex. However, after cuprizone diet cessation, which promotes remyelination, synaptic transmission is recovered (Das et al., 2020; Wellman et al., 2020). This suggests that remyelination has the potential to improve nerve conduction, but beckons the question of whether the axon itself recovers following chronic demyelination. Crawford et al. (2009) demonstrated a critical window for repair after demyelination, after which time axonal function cannot fully recover. These data were consistent with sustained axonal injury and decreased efficacy of remyelination. Taken together, these data emphasize a critical period for remyelination suggesting that early intervention of regenerative strategies is necessary to preserve axonal integrity.

Chronically demyelinated axons are subjected to environmental insult from ongoing inflammation, leading to axonal transection which is observed in both MS and EAE (Nikić et al., 2011; Trapp et al., 1998). Axon segments distal to the transected portion also eventually degrade by Wallerian degeneration (Kaneko et al., 2006; Singh et al., 2017). With prolonged demyelination comes damage to the axon itself, leading to accumulation and progression of neurological disability. While mechanisms of neurodegeneration independent of demyelination may occur in MS, the prominent effects of demyelination leading to axonal degeneration make remyelination an attractive neuroprotective and therapeutic strategy.

### 3.3 Neuroprotection and remyelination

The myelinating oligodendrocyte is targeted during adaptive immune responses in MS. Oligodendrocyte progenitor cells (OPCs) proliferate in response to inflammation and are found in MS lesions (Lucchinetti et al., 1999; Raine et al., 1981), but fail to fully differentiate and efficiently remyelinate axons (Kuhlmann et al., 2008). While some remyelination occurs and leads to the remission of symptoms in RRMS, over time this process misses the window of recovery and cannot overcome the accumulating axonal damage (reviewed by (Franklin, 2002; Franklin & ffrench‐Constant, 2017; Stangel et al., 2017). A study by Jäkel et al. (2019) showed by single nuclei RNAseq from *post‐mortem* MS brains that there are changes in sub‐clusters of oligodendrocytes and OPCs that may contribute to the general inability to efficiently remyelinate. Studies using models of demyelination and remyelination have therefore been performed to better understand how to improve remyelination potential and axonal recovery.

One method to reduce demyelination and axonal injury is to protect the oligodendrocyte during neuroinflammation. One significant cause of neuronal and oligodendroglial cell death is an abundance of intracellular calcium triggered by overactivation of ionotropic glutamate receptors. The AMPA‐type ionotropic glutamate receptor in particular renders oligodendrocytes particularly susceptible to this phenomenon, termed “excitoxicity” (reviewed by [Centonze et al., 2010; Macrez et al., 2016]). Global knockouts of AMPA‐type glutamate receptor subunits or pharmacological AMPA receptor antagonists in EAE have ameliorated disease severity in mice (Bannerman et al., 2007; Pitt et al., 2000; Smith et al., 2000). Specifically, Evonuk et al. (2020) showed that by inducibly deleting the GluA4 subunit from mature oligodendrocytes prior to EAE, there was a reduction in clinical symptoms, demyelination, and loss of myelinated axons. This suggests that by protecting oligodendrocytes from cell death, axons which they myelinate are also protected. Methods for reducing excitotoxic cell death have been investigated in MS, including AMPA and NMDA receptor antagonists and glutamate scavenging methods (Gottlieb et al., 2003; Levite, 2017; Macrez et al., 2016). Since glutamate is the most abundant excitatory neurotransmitter in the CNS, the application of these techniques has been complicated for the clinical treatment of MS. Nonetheless, these studies highlight that axonal protection can be achieved by promoting the integrity of the oligodendrocyte, which has also been the aim of remyelinating strategies.

Another barrier to remyelination outside of direct effects of the inflammatory milieu is cellular senescence. It has been previously shown that failed OPC recruitment and differentiation in general contributes to remyelination failure in chronic MS (Boyd et al., 2013; Kuhlmann et al., 2008). OPC differentiation is, in part, dependent upon macrophage and microglial clearance of mature myelin debris, as myelin itself can inhibit OPC differentiation (Baer et al., 2009; Kotter et al., 2006). A study by Ruckh et al. (2012) showed that remyelination following lysolecithin‐induced demyelination is enhanced by promoting OPC proliferation and differentiation in aged mice when stimulated with young macrophages by parabiosis. Additionally, this study showed an increased clearance of myelin debris in aged mice following demyelination when exposed to the macrophages from the young mice (Ruckh et al., 2012). Markers of senescence have also been observed in neural progenitor cells and OPCs that contribute to impaired remyelination in progressive MS and mouse models (Nicaise et al., 2019; Sim et al., 2002). A study by Neumann et al. (2019) aimed to overcome the effects of aging on the regenerative capacity of OPCs through treatment with metformin, a fasting mimetic. Their results showed both in vitro and with experimental demyelination using ethidium bromide in rats that metformin treatment enhanced OPC differentiation, remyelination, and increased mitochondrial metabolic function (Neumann et al., 2019). Together these data suggest that axonal degeneration is a prominent contributor to progressive decline in MS, and that reducing demyelination and promoting early remyelination can protect myelinated axons, restore neuronal function, and prevent disease progression.

## 4 CONCLUSIONS

Multiple sclerosis is a highly variable disease with the onset of neurodegeneration occurring at the earliest stages, accelerated with disease activity, and likely continuing insidiously throughout disease courses. Mechanisms underlying this disease pathology and the heterogeneity of clinical courses in patients are still unclear, warranting research to elucidate pathways of degeneration and the potential for repair. Although historically MS has been studied in the cerebral white matter, as it is a frequent site of inflammatory lesions, it does not explain disability or a transition to progressive MS completely. While DMTs reduce inflammatory relapses, therapies to prevent axonal damage and progressive disease are needed. Brain and spinal cord atrophy is a visible indicator of neurodegeneration and deep gray matter and spinal cord involvement more closely ties with disease progression (Arrambide et al., 2018; Azevedo et al., 2018; Bischof et al., 2021; Calabrese et al., 2007). Gray matter pathology including cortical lesions separate from the white matter have also been a better predictor of disease progression than global lesion burden (Calabrese et al., 2012; Mahajan et al., 2020). While being able to predict disease progression is important and can aid in determining course of action from a therapeutic standpoint, the underlying mechanisms behind these clinical findings remain unclear. It is therefore with a better understanding of neurodegenerative mechanisms that new therapeutic strategies for neuroprotection can be defined.

There are several key processes that are altered early in disease due to the inflammatory nature of MS that ultimately lead to irreversible axonal and neuronal loss. Ongoing inflammation and demyelination contributes to a cycle of oxidative stress and mitochondrial dysfunction (Sorbara et al., 2014). Mutations in mitochondrial DNA have been identified in MS (Dutta et al., 2006), and animal models have supported early deficits in mitochondrial transport during EAE before demyelination (Nikić et al., 2011). This supports that the inflammatory milieu can cause primary axonal dysfunction, which is further exacerbated by chronic demyelination leading to axonal transection and degeneration in MS. Other models have shown that there is a small window of opportunity in which axons can be remyelinated and their function restored following demyelination (Crawford et al., 2009). Glutamate dysregulation has also been shown to be elevated in early MS and is a predictor for new inflammatory activity. Activated immune cells release glutamate, and the inflammatory environment results in dysregulation of various glutamate transporters and receptors in the CNS (Macrez et al., 2016). This can lead to excitotoxic cell death of both neurons and oligodendrocytes (Centonze et al., 2010; Pitt et al., 2000). Protecting mature oligodendrocytes from excitotoxicity leads to reduced demyelination *and* protection against myelinated axon loss in the EAE model (Evonuk et al., 2020). This suggests that specific protection of oligodendrocytes can help protect axons during neuroinflammation. However, remyelination cannot occur without appropriate cellular signaling and support. Aging and cellular senescence prevent OPC differentiation and maturation (Ruckh et al., 2012), presenting another barrier to recovery outside of glial scarring and neurodegeneration itself. Together these data emphasize the multifaceted nature of MS pathology, and that targeting oligodendrocyte and axonal metabolic health may be a critical strategy early in disease to protect against future neurodegeneration and progressive decline.

## AUTHOR CONTRIBUTIONS

**Gabrielle Mey:** Conceptualization (supporting); writing – original draft (supporting). **Kedar Mahajan:** Conceptualization (supporting); writing – original draft (supporting); writing – review and editing (supporting). **Tara DeSilva:** Conceptualization (lead); funding acquisition (lead); resources (lead); supervision (lead); validation (lead); writing – review and editing (lead). The authors declare no competing interests.

## Data Availability

Data sharing is not applicable to this article as no new data were created or analyzed in this study.
